# DEAD-box RNA helicase protein DDX21 as a prognosis marker for early stage colorectal cancer with microsatellite instability

**DOI:** 10.1038/s41598-020-79049-9

**Published:** 2020-12-16

**Authors:** Atsushi Tanaka, Julia Y. Wang, Jinru Shia, Yihua Zhou, Makiko Ogawa, Ronald C. Hendrickson, David S. Klimstra, Michael H. Roehrl

**Affiliations:** 1 Department of Pathology, Memorial Sloan Kettering Cancer Center, New York, NY USA; 2 Department of Pathology, Graduate School of Medicine, University of Tokyo, Tokyo, Japan; 3 Human Oncology and Pathogenesis Program, Memorial Sloan Kettering Cancer Center, New York, NY USA; 4 Curandis, New York, NY USA; 5 ICU Department, Second Affiliated Hospital of Nanchang University, Nanchang, Jiangxi China; 6 Sloan Kettering Institute, Memorial Sloan Kettering Cancer Center, New York, NY USA

**Keywords:** Gastrointestinal cancer, Cancer, Gastrointestinal diseases, Biomarkers, Oncology, Translational research

## Abstract

DEAD-box RNA helicase DDX21 (also named nucleolar RNA helicase 2) is a nuclear autoantigen with undefined roles in cancer. To explore possible roles of autoimmune recognition in cancer immunity, we examined DDX21 protein expression in colorectal cancer tissue and its association with patient clinical outcomes. Unbiased deep proteomic profiling of two independent colorectal cancer cohorts using mass spectrometry showed that DDX21 protein was significantly upregulated in cancer relative to benign mucosa. We then examined DDX21 protein expression in a validation group of 710 patients, 619 of whom with early stage and 91 with late stage colorectal cancers. DDX21 was detected mostly in the tumor cell nuclei, with high expression in some mitotic cells. High levels of DDX21 protein were found in 28% of stage I, 21% of stage II, 30% of stage III, and 32% of stage IV colorectal cancer cases. DDX21 expression levels correlated with non-mucinous histology in early stage cancers but not with other clinicopathological features such as patient gender, age, tumor location, tumor grade, or mismatch repair status in any cancer stage. Kaplan–Meier analyses revealed that high DDX21 protein levels was associated with longer survival in patients with early stage colorectal cancer, especially longer disease-free survival in patients with microsatellite instability (MSI) cancers, but no such correlations were found for the microsatellite stable subtype or late stage colorectal cancer. Univariate and multivariate analyses also identified high DDX21 protein expression as an independent favorable prognostic marker for early stage MSI colorectal cancer.

## Introduction

Colorectal cancer is a leading cause of death among cancer patients. Increasingly, colorectal cancer is recognized as a heterogeneous disease that is characterized by accumulation of genetic and epigenetic alterations affecting distinct protein pathways^1^. Risk assessment of patients with early stage colorectal cancer is particularly critical because it provides guidance for choosing the best curative cancer treatment strategy for an individual patient^2^. A number of genetic markers such as *NRAS*, *KRAS*, and *BRAF* are used in cancer risk stratification^3,4^. The DNA mismatch repair status classifies colorectal cancers into either microsatellite stability (MSS) or microsatellite instability (MSI) subtypes^1^. Given the fact that targeted cancer therapies, including immunotherapy, all directly target cellular proteins in particular pathways, not genes, there is a need for protein-based molecular biomarkers with an underlying functional pathophysiologic rationale. Such functional protein markers have the potential to offer more precise prognostic and predictive value, which would enable better risk stratification of early stage cancer after surgical removal and better selection of patients for adjuvant chemotherapy or immunotherapy, while avoiding overtreatment or immune-related adverse events^5,6^.

We used unbiased deep proteomics by mass spectrometry to define proteomic changes that occur in colorectal cancer and to identify specific proteins that may serve as biomarkers^7–10^. We are especially interested in studying proteins that are known to be antigenic to the human immune system because we hypothesize that these proteins may induce diagnostically useful antibodies and serve as predictors of immunotherapy response in patients^9,10^. In this paper, we focus on the discovery and characterization of DEAD-box RNA helicase DDX21 in colorectal cancer since function and clinical significance of DDX21 in cancers, including survival effects, are largely unknown. We validated DDX21 protein expression in a large cohort of patients in order to evaluate the clinical prognostic significance of DDX21 in colorectal cancer, especially its potential as a prognostic marker for early stage cancer.

## Materials and methods

### Clinical specimens and pathological data

Adenocarcinoma tissues and normal benign colonic mucosa were obtained from the Precision Pathology Biobanking Center of Memorial Sloan Kettering Cancer Center. The study was approved by the Institutional Review Board of Memorial Sloan Kettering Cancer Center. Clinical data were acquired retrospectively and in an anonymized manner such that patient consent was not required (as determined by the MSKCC Institutional Review Board). All methods were performed in accordance with relevant guidelines and regulations. Clinical data, including patient demographics, treatment history, recurrence status, and MMR status, were retrieved from medical records. Histologic type and other pathological parameters were extracted from diagnostic pathology reports, and diagnoses, tumor content, and tumor purity for all samples were verified by gastrointestinal subspecialty pathologists (AT and MHR). Counts of tumor infiltrating lymphocytes (TILs) per 10 high-power fields (40 × objective) were retrieved from our research database for the tissue microarray cohort.

### Fresh frozen tissue selection

For the initial proteomic discovery of protein biomarkers, we selected and studied two independent cohorts of fresh frozen tissues: one cohort of 22 CRC cases and a second cohort of 15 CRC cases. All tissue samples fulfilled the sample criteria of high tumor content (> 50%) or benign normal mucosa (for matched normal samples), minimal gross and microscopic necrosis (< 5%), and low blood contamination (< 5%). Matched pairs of frozen tumor tissue and benign colonic mucosa harvested away from the cancer (carefully stripped without muscularis propria) were retrieved from the vapor phase liquid nitrogen repository.

### Tissue proteome extraction

Similar to our prior work^7,8,11^, samples of 5 mg of frozen tissue were thawed on ice and lysed with 200 μl lysis buffer containing 8 M urea, 0.1 M ammonium bicarbonate, phosphatase inhibitors 2 and 3 (Sigma), and protease inhibitors (Roche). The tissue mixture was homogenized with 12 cycles of 1-min sonication at 120 W power (FB120, Fisher Scientific) and intermittent cooling. After centrifugation at 14,000*g* for 30 min at 4 °C, the supernatant containing all soluble proteins was collected. The protein concentration was determined by a BCA assay (Pierce), and extracted proteomes were stored at − 80 °C until further analysis.

### In-solution protein digestion

Aliquots of 50 µg of proteome lysate were reduced with 5 mM dithiothreitol at 56 °C for 30 min and then cooled to room temperate^7,8,11^. The reduced proteins were alkylated with 11 mM iodoacetamide at room temperature for 30 min in the dark. The protein solution was diluted sixfold with 50 mM ammonium bicarbonate and digested with trypsin and Lys-C (0.2 μg/μl, both from Promega) at 1:50 (w/w) at 37 °C for 12 h. The digestion was stopped by the addition of trifluoroacetic acid to a final concentration of 1%. The mixture was centrifuged at 14,000*g* for 10 min at room temperature. The clear supernatant was collected and desalted on a C_18_ StageTip (lab-made). Desalted peptides were dried in a SpeedVac vacuum concentrator and re-dissolved in 10–15 μl of 3% acetonitrile/0.1% formic acid and stored at − 20 °C.

### Proteomic analysis

Desalted peptides, approximately 1 μg, were injected into a 50-cm C_18_ capillary column mounted to an Easy-nLC 1200 system coupled to an Orbitrap Fusion Lumos mass spectrometer (Thermo Scientific)^7,8,11^. Peptides were eluted over a 200-min gradient in 2–35% buffer B (0.1% (v/v) formic acid, 100% acetonitrile) and buffer A (0.1% formic acid, 100% HPLC-grade water) at a flow rate of 300 nl/min. MS data were acquired with an automatic switch between a full scan and 10 data-dependent MS/MS scans. The target value for full-scan MS spectra was 1 × 10^6^ charges in the 375–1500 *m/z* range with a maximum injection time of 50 ms and a resolution of 60,000 at 200 *m/z* in profile mode. Isolation of precursors was performed with a window of 1.4 *m/z*. Precursors were fragmented by higher-energy C-trap dissociation with a normalized collision energy of 30 eV. MS/MS scans were acquired at a resolution of 15,000 at 200 *m/z* with an ion target value of 5 × 10^4^, maximum injection time of 100 ms, and dynamic exclusion for 15 s in centroid mode.

### Protein sequencing data analysis

We applied an overall data analysis strategy that is based on prior work from our laboratory^7–11^. Briefly, label-free protein quantification was carried out with MaxQuant (version 1.6.4) and the Andromeda search engine^12,13^. The first and the main maximum precursor mass tolerances were set to 20 and 6 ppm, respectively. The reference human proteome database was downloaded from UniProt. The search assumed trypsin and Lys-C digestions with up to 2 missed cleavages. A minimum of 1 peptide was required for protein identification, but 2 peptides were required to calculate a protein level ratio. The modifications used as variable modifications for protein identification and quantification included oxidation of methionine, acetylation of the protein N-terminus, phosphorylation of serine, threonine, and tyrosine residues, and deamidation of glutamine and asparagine. Significantly up-regulated and down-regulated proteins were identified with Perseus software (version 1.6.5)^14,15^. The mass spectrometry proteomics data have been deposited to the ProteomeXchange Consortium via the PRIDE partner repository with the dataset identifiers PXD019103 and PXD019504.

### Tissue microarrays

For tissue microarrays^7,8,11^, formalin fixed and paraffin embedded tissue blocks from 710 colorectal cancer patients were selected (with no patient overlap with the two frozen tissue cohorts used for mass spectrometry). Three separate 2-mm tissue cores from each tumor case were drilled out from each donor paraffin block and transferred to tissue array blocks using a robotic TMA arrayer (TMA Grand Master, 3DHistech). Tumor and normal areas were selected based on rigorous review of individual histologic slides for each donor block and electronic image-based coring target area selection in the TMA Grand Master software.

### Immunohistochemistry (IHC)

Formalin-fixed paraffin-embedded tissues were cut into 4-μm sections^7,8,11^. Paraffin was removed with xylene, and antigens were retrieval by heat-mediated epitope retrieval (pH 6.0). DDX21 expression was determined with DDX21-specific polyclonal antibodies (HPA036593, 1:200 dilution, Atlas Antibodies). IHC staining was conducted with Leica BOND-MAX automation. We assessed DDX21 nuclear staining as positive if 10% or more tumor cells showed nuclear staining. Assessment of all tissue samples was independently performed by two pathologists without any clinical information. In cases of discrepancies in immunohistochemical assessment between the two pathologists, the cases were reviewed by them together and a consensus score was determined.

### TCGA data

We analyzed mRNA sequencing data and clinical information from the TCGA (cohort of 244 colorectal cancers^16^) by accessing the cBioPortal for Cancer Genomics (https://www.cbioportal.org/). We also studied a 597-case TCGA colorectal cancer cohort^17^ that lacks comprehensive clinical stage, *KRAS* mutation status, and mismatch repair status annotation.

### Statistical analyses

Similar to prior work from our laboratory^7,8,11^, categorical variables were compared using Fisher’s exact test. Numerical values were analyzed by the Mann–Whitney U test. Survival analyses were performed using the Kaplan–Meier method and compared by a log-rank test. Multivariate analyses of prognostic factors were performed with logistic regression models by using factors that showed significant differences in univariate analyses (*p* < 0.05). Statistical analyses were performed with the JMP Pro 14 software (SAS). All statistical analyses were considered significant with *p* < 0.05.

## Results

### Proteomic analysis of colorectal cancer and identification of DDX21

**Figure 1:**
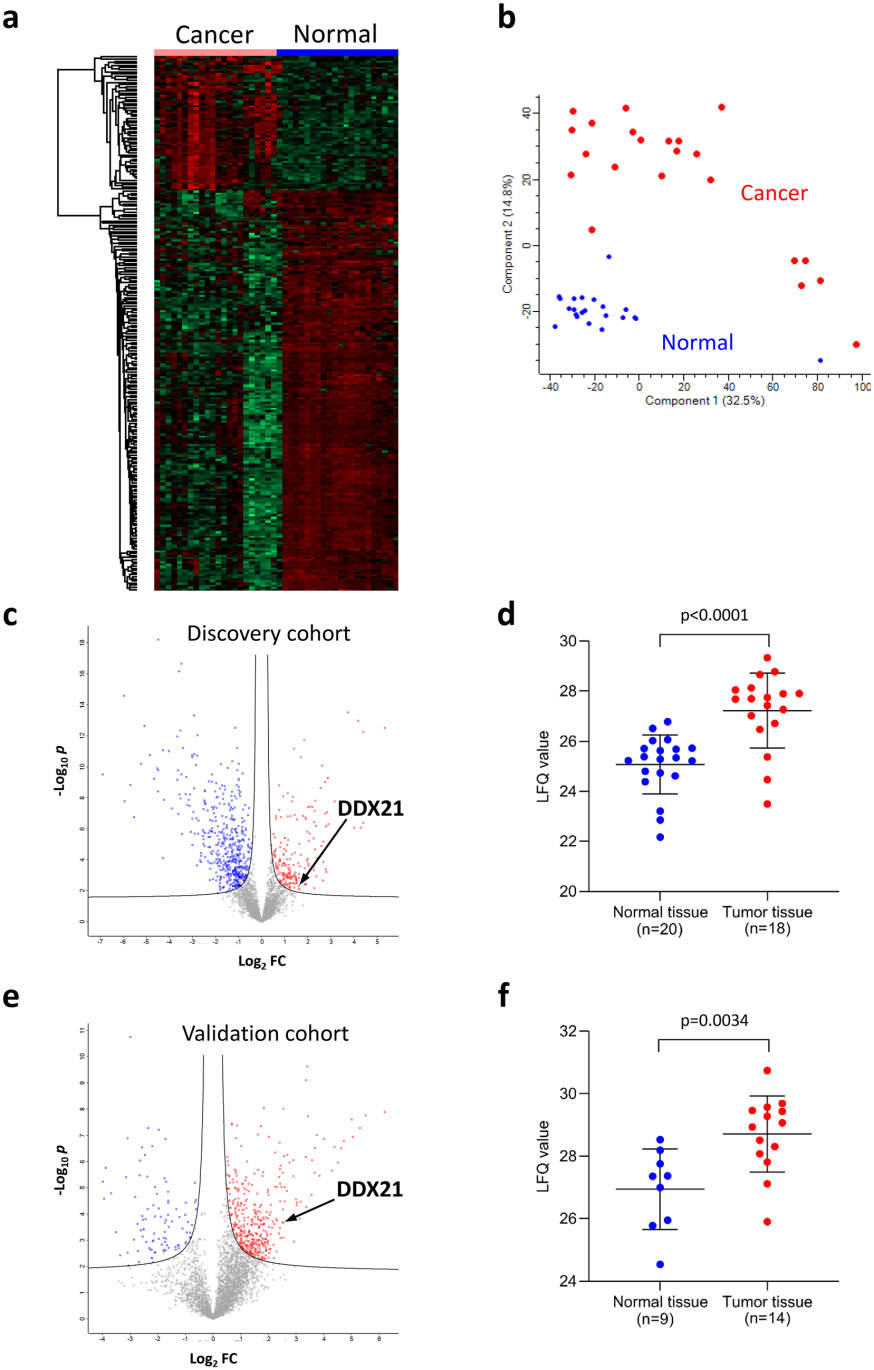
Mass spectrometric proteomic characterization of DDX21 in colorectal cancer. (**a**) Heat map of proteome differences between colorectal cancer and benign colonic mucosa for the initial discovery cohort of 22 patients. Clear separation of cancer vs. benign normal mucosa is observed (left vs. right). A total of 251 proteins are shown (from top to bottom) with ≥ threefold abundance change (63 up- and 188 down-regulated) in cancer vs. normal. Shades of red denote up-regulation and shades of green denote down-regulation relative to the matched comparison. (**b**) Principal component analysis of the initial discovery cohort of 22 patients based on 3426 proteins from colorectal cancer vs. benign normal colonic mucosa as measured by mass spectrometry (corresponding to the set of all shared protein identifications quantifiable across ≥ 40% of all samples). Percentages in parentheses indicate percentage of variance explained by the respective eigenvector (principal component). (**c**) Volcano plot of relative abundances of 3426 proteins from colorectal cancer vs. benign normal colonic mucosa as measured by mass spectrometry in matched samples from the initial discovery cohort of 22 patients. The DDX21 protein is highlighted by the arrow. The hyperbolic solid lines show the false discovery rate frontier set to 0.01. The x-axis shows the log_2_ of the fold change (FC) of protein abundance (ratio of cancer to benign mucosa). The y-axis shows the negative log_10_ of the t-test *p* value for a particular protein (dot in the volcano plot). (**d**) Label-free quantification (LFQ) of DDX21 protein by mass spectrometry in the initial discovery cohort (only samples where LFQ values could be calculated by MaxQuant from mass spectra are shown). Horizontal line segments denote distribution mean and ± 1 standard deviation, respectively. Statistical comparison by Mann–Whitney U test. (**e**) Volcano plot of relative abundances of 3501 proteins from colorectal cancer vs. benign colonic mucosa as measured by mass spectrometry in matched samples from the independent validation cohort of 15 patients. DDX21 protein is highlighted by the arrow. The hyperbolic solid lines show the false discovery rate frontier set to 0.01. The x-axis shows the log_2_ of the fold change (FC) of protein abundance (ratio of cancer to benign mucosa). The y-axis shows the negative log_10_ of the t-test *p* value for a particular protein (dot in the volcano plot). (**f**) Label-free quantification (LFQ) of DDX21 protein by mass spectrometry in the independent validation cohort (only samples where LFQ values could be calculated by MaxQuant from mass spectra are shown). Horizontal line segments denote distribution mean and ± 1 standard deviation, respectively. Statistical comparison by Mann–Whitney U test.

We first examined a group of 22 patients whose primary colorectal cancer and matched benign mucosa had been freshly frozen in liquid nitrogen. Using deep unbiased tissue proteomics by Fourier transform mass spectrometry^7–10^, we found that proteomic profiling robustly separates colorectal cancer from benign mucosa (Fig. 1a,b). We then searched specifically for proteins that (i) were upregulated in cancer vs. benign mucosa and that (ii) are also known human autoantigens based on literature searches. This approach led us to the identification of DDX21 (Fig. 1c,d). Although DDX21, also termed nucleolar RNA helicase 2, is a known autoantigen, with autoantibodies found in patients with connective diseases and gastric antral vascular ectasia (watermelon stomach disease)^18–20^, its clinical significance in colorectal cancer, including effects on outcome, is unknown. This motivated us to study DDX21 in colorectal cancer. For validation, we repeated unbiased proteomic profiling using a second (independent) cohort of 15 patients. DDX21 protein was again found to be significantly overexpressed in cancer relative to matched benign mucosa (Fig. 1e,f).

### DDX21 protein expression patterns in colorectal cancer tissues

We investigated DDX21 protein expression in 710 colorectal cancer cases using tissue microarrays, which included a large cohort of 619 patients with early stage (stages I and II) cancer (Table 1) and a cohort of 91 patients with late stage (stages III and IV) cancer (Table 2). Because prognostic markers of early stage cancers are especially valuable for clinical cancer management, we carefully assembled this large cohort of early stage cancer cases with a representative distribution of colorectal cancers, comprised of 319 male and 300 female patients. The late stage cancer cohort is smaller and included 45 male and 46 female patients.

**Table 1:** DDX21 protein expression in the early stage colorectal cancer cohort (UICC/AJCC stages I and II combined).

DDX21 protein	Negative (n = 475)	Positive (n = 144)	p value*
Gender			> 0.9999
Male	245	74	
Female	230	70	
Age			0.7723
≤ 65	200	58	
> 65	275	86	
Histology			**0.0391**
Mucinous	46	6	
Non-mucinous	429	138	
Tumor differentiation			0.1667
G1/G2	440	128	
G3	35	16	
Tumor location			0.2964
Left	242	66	
Right	233	78	
UICC/AJCC stage			**0.0375**
I	161	63	
II	314	81	
MMR status			0.3646
MSS	371	107	
MSI	104	37	

p values < 0.05 are indicated in bold

MMR, mismatch repair.

*Fisher’s exact test.

**Table 2:** DDX21 protein expression in the late stage colorectal cancer cohort (UICC/AJCC stages III and IV combined).

DDX21 protein	Negative (n = 63)	Positive (n = 28)	p value*
**Gender**			0.6540
Male	30	15	
Female	33	13	
**Age**			0.2446
≤ 65	59	24	
> 65	4	4	
**Histology**			0.2655
Mucinous	8	1	
Non-mucinous	55	27	
**Tumor differentiation**			> 0.9999
G1/G2	55	25	
G3	8	3	
**Tumor location**			0.6429
Left	40	16	
Right	23	12	
**UICC/AJCC stage**			> 0.9999
III	46	20	
IV	17	8	
**MMR status**			0.7487
MSS	54	25	
MSI	9	3	

MMR, mismatch repair.

*Fisher’s exact test.

**Figure 2:**
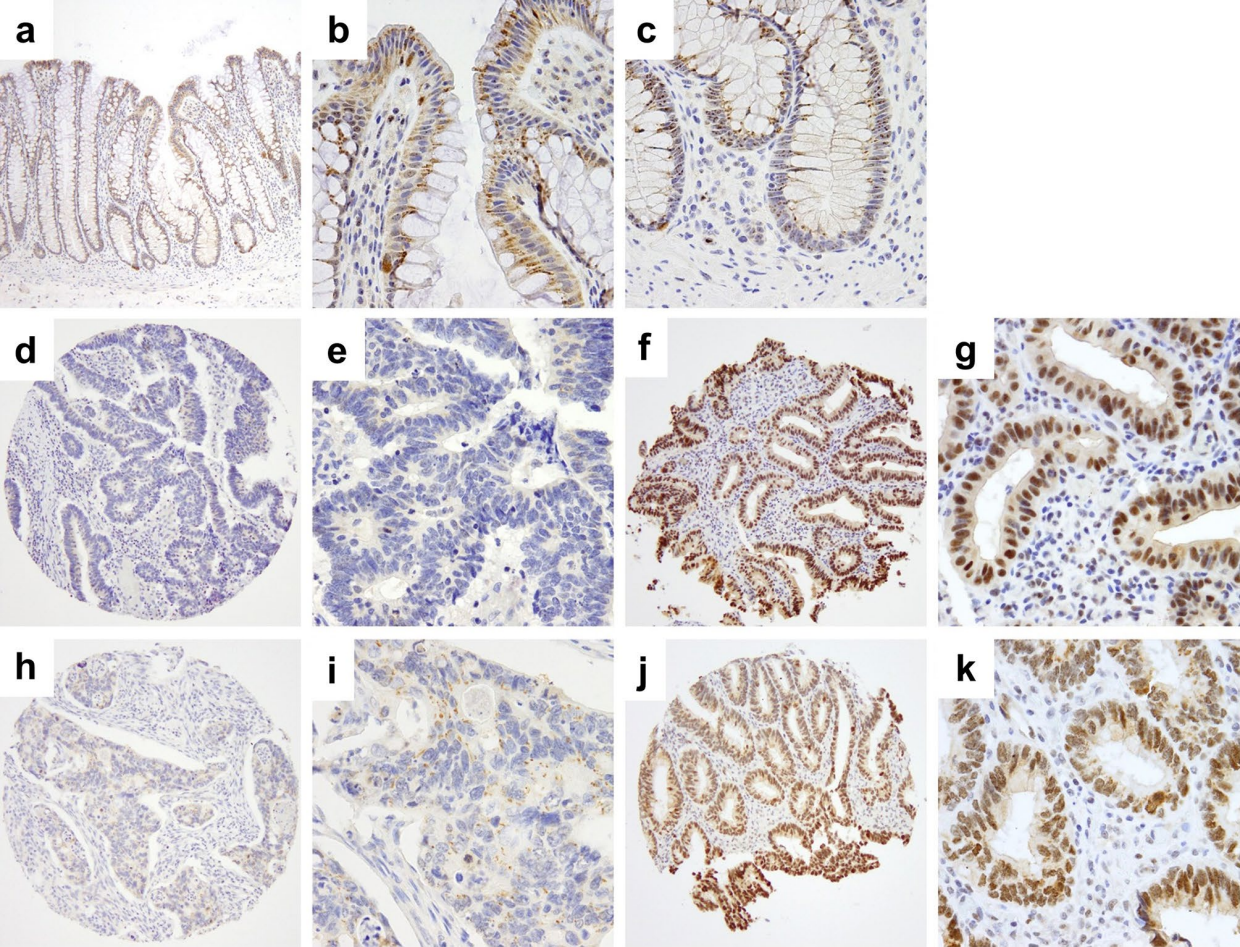
Representative DDX21 protein detection by immunohistochemistry of TMA cores. (**a-c**) Benign colonic mucosa with negative or weak cytoplasmic or weak nucleolar staining for DDX21 in enterocytes. (**d**,**e**) Representative early stage colorectal cancer with negative staining for DDX21. (**f**,**g**) Representative early stage colorectal cancer with strongly positive nuclear DDX21 staining in cancer cells. Note the absence of staining in stromal cells of the lamina propria. (**h**,**i**) Representative late stage colorectal cancer with negative staining for DDX21. (**j**,**k**) Representative late stage colorectal cancer with strongly positive nuclear DDX21 staining in cancer cells. Note the absence of staining in stromal cells of the lamina propria. Original magnifications: ×20 (**a**,**d**,**f**,**h**,**j**) and ×200 (**b**,**c**,**e**,**g**,**i**,**k**).

**Figure 3:**
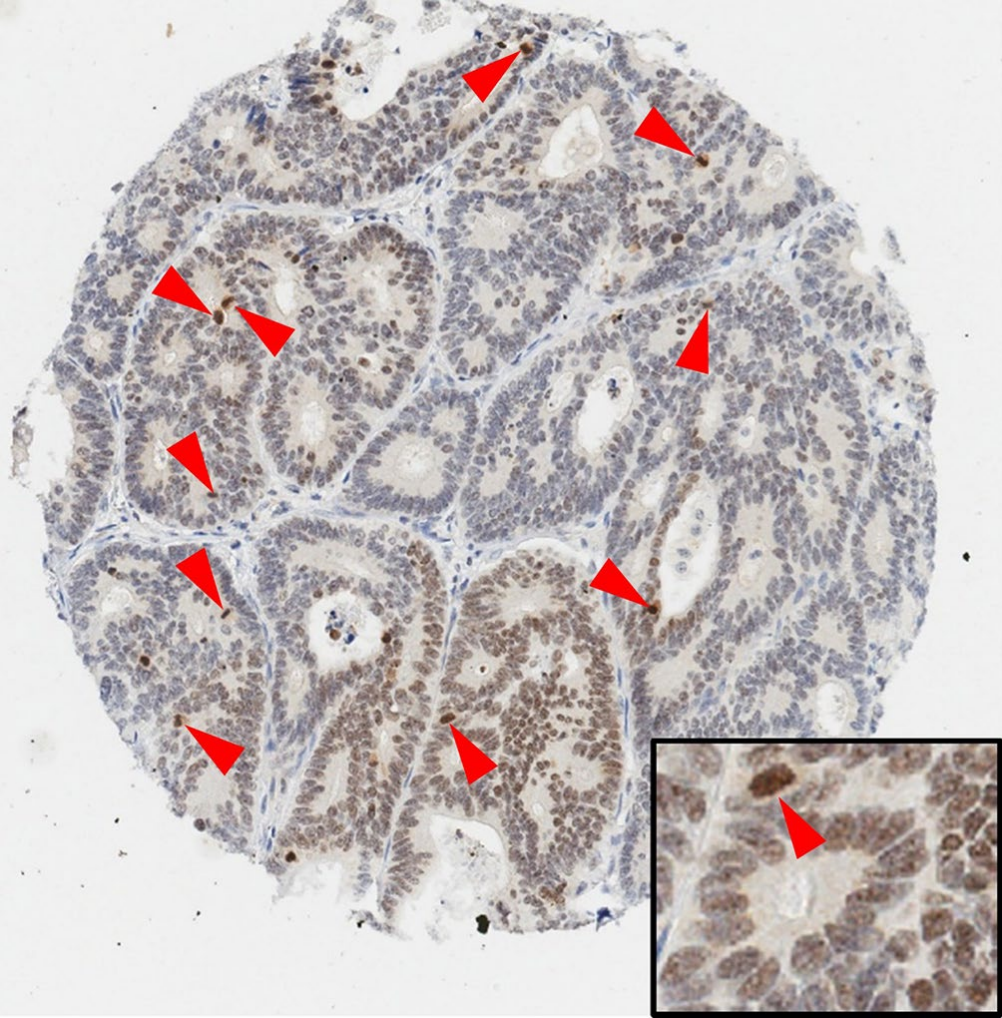
Representative image of a case where DDX21 protein expression appears strongly associated with mitotic cells, localized to condensed chromosomal structures (red arrowheads). Original magnifications: ×20 (main image), ×400 (insert).

Expression of DDX21 protein in tissue was examined by immunohistochemical detection. In normal benign colonic mucosa, protein expression of DDX21 was barely detectable with at most weak cytoplasmic and weak to undetectable nuclear (then restricted to nucleolar) DDX21 (Fig. 2a–c). Cancers fell into two categories (with little heterogeneity within a case): (i) essentially negative or very weak cytoplasmic DDX21 (examples of an early stage and a late stage cancer in Fig. 2d,e,h,i, respectively) or (ii) strong (predominantly nuclear) staining for DDX21 (examples of an early stage and a late stage cancer in Fig. 2f,g,j,k, respectively). In about 20–30% of colorectal cancer cases, DDX21 overexpression was detected in cancer cells. Stromal cells of the lamina propria were essentially negative for detectable DDX21 protein (Fig. 2). Interestingly, DDX21 protein expression was particularly high in some mitotic cells (Fig. 3), a feature shared with other intrinsic autoantigenic proteins^21–24^.

### DDX21 protein expression and clinicopathological features of colorectal cancer

To explore associations between DDX21 protein expression and clinicopathological features of colorectal cancer, we evaluated the expression level of DDX21 in each tissue in a semi-quantitative fashion. Each tissue sample was scored independently by two expert pathologists to obtain unbiased readings. Based on this scoring, the cancer cohort was then divided into two groups, a positive (or high expression) group that showed positive nuclear DDX21 staining in ≥ 10% of cancer cells, and a negative (or low expression) group that showed negative staining or staining in < 10% of cancer cells.

In the early stage colorectal cancer cohort, positive DDX21 expression was detected in 28.1% (63/224) of stage I and 20.5% (81/395) of stage II patients (Table 1). DDX21 expression appeared to be more prevalent in stage I than in stage II cancer tissues, with the difference being statistically significant (Table 1). There appeared to be no difference in DDX21 expression in the late stage colorectal cancer cohort, as positive DDX21 expression was detected in 30.3% (20/66) of stage III and 32.0% (8/25) of stage IV patients (Table 2).

DDX21 expression levels were then compared with various clinicopathological features (Tables 1, 2). In the early stage cohort, DDX21 expression levels did not differ significantly with regard to patient gender, patient age, tumor differentiation, tumor location, or mismatch repair status. However, positive DDX21 expression was significantly more prominent in non-mucinous carcinoma (32.2%) vs. mucinous carcinoma (13.0%) (Table 1). In the late stage cohort, DDX21 expression showed no significant association with any of the examined clinicopathological features, including patient gender or age, tumor differentiation or stage, mucinous histology, tumor location, or mismatch repair status (Table 2).

As DDX21 is a known nuclear autoantigen^2,19,20^, we investigated whether DDX21 expression correlates with the density (cell count per 10 high-power fields) of tumor infiltrating lymphocytes (TILs). Among 710 cases of the TMA cohort, 230 cases had available TIL data (113 early-stage CRCs and 117 late-stage CRCs). Neither MSS CRCs nor MSI CRCs showed significant TIL differences between DDX21 high and low groups (Supplementary Fig. 1).

### Correlation between DDX21 protein expression and patient survival

**Figure 4:**
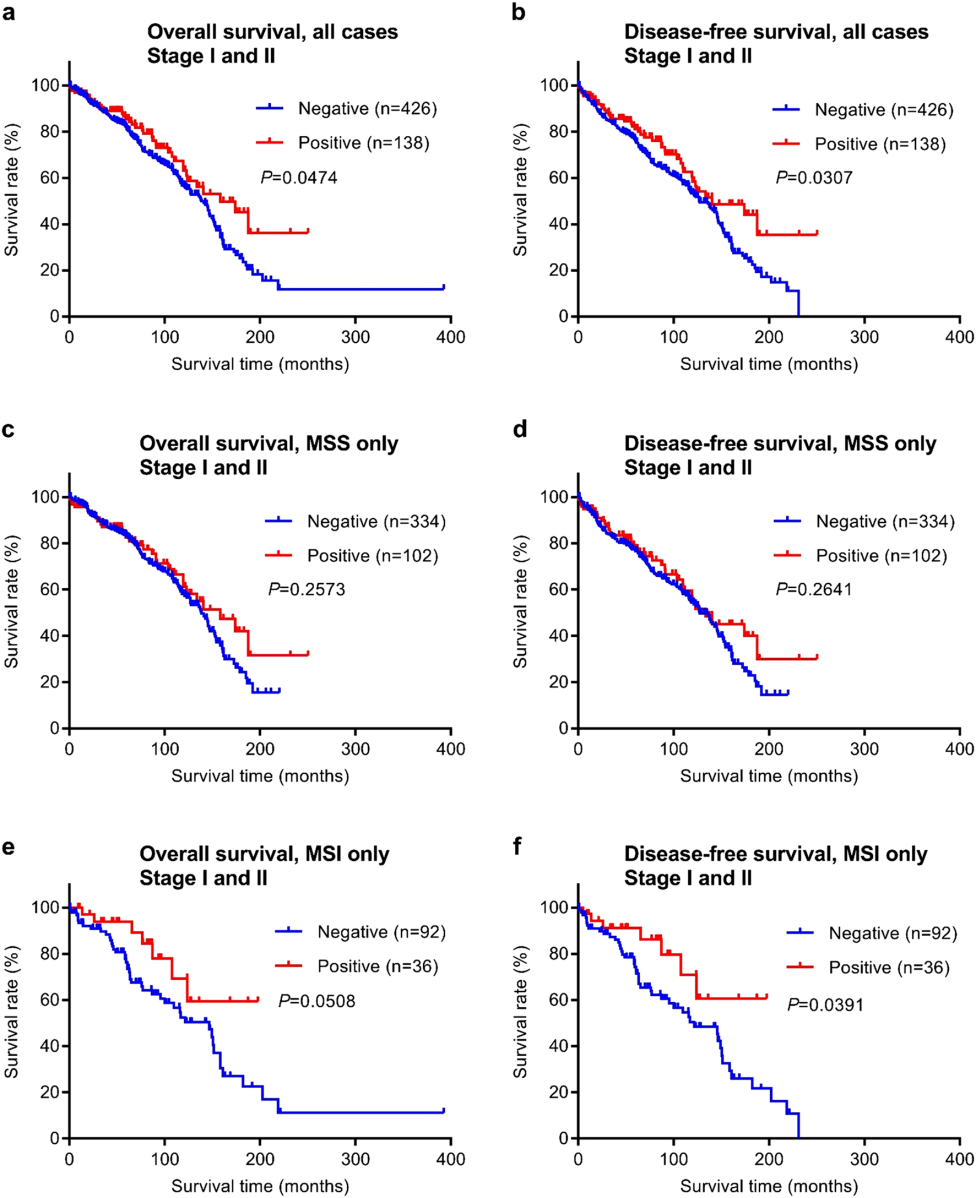
Overall survival and disease-free survival analysis of early stage (stages I and II) CRC stratified by DDX21 protein expression. (**a**,**b**) Kaplan–Meier curves with stratification by DDX21 protein expression for all cases (n = 564). (**c**,**d**) Kaplan–Meier curves for MSS CRC cases only (n = 436). (**e**,**f**) Kaplan–Meier curves for MSI CRC cases only (n = 128).

**Figure 5:**
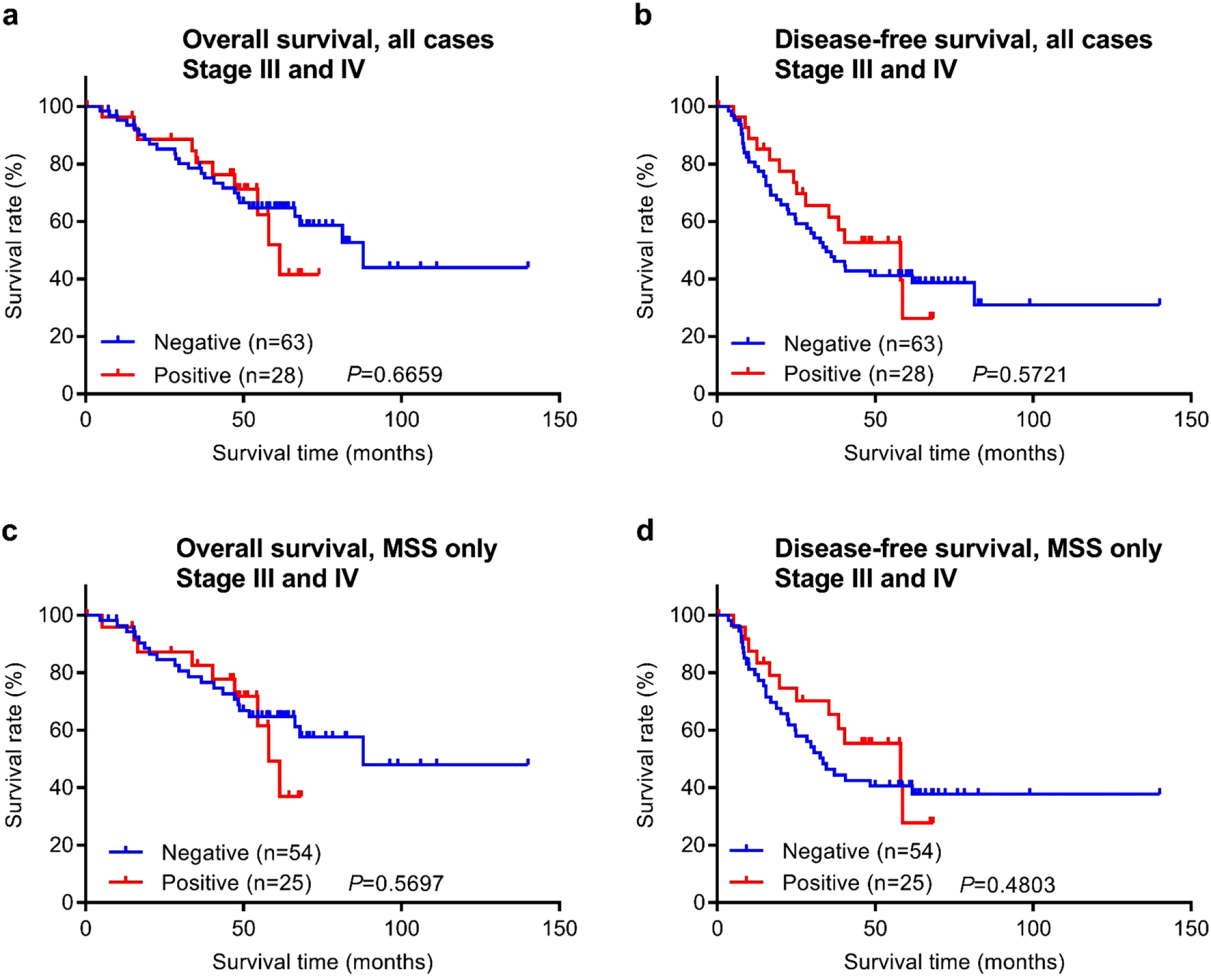
Overall survival and disease-free survival analysis of late stage (stages III and IV) CRC stratified by DDX21 protein expression. (**a**,**b**) Kaplan–Meier curves with stratification by DDX21 protein expression for all cases (n = 91). (**c**,**d**) Kaplan–Meier curves for MSS CRC cases only (n = 79). Note that Kaplan–Meier statistics for MSI CRC cases only were not meaningful and are thus not shown due to the small number of cases in that group (n = 12).

To evaluate the prognostic potential of DDX21 protein expression for colorectal cancer, we investigated the relationship between patient survival times and DDX21 using Kaplan–Meier analyses. Both the overall survival time and the disease-free survival time were analyzed. The stage I and II patients of this study had been followed for a range of 0.2–392.5 months, with a mean follow-up time of 80.6 months and a median follow-up time of 72.5 months (Fig. 4). The stage III and VI patients of this study had been followed for a range of 0.4–140 months, with a mean follow-up time of 51.2 months and a median follow-up time of 53.3 months (Fig. 5).

Of the 619 early stage cases examined by immunohistochemistry, 564 cases had evaluable survival follow-up data available in the medical records. Importantly, these 564 cases had no adjuvant chemotherapy history, and thus this group serves as an ideal unbiased cohort for prognostic outcome studies. When all 564 early stage cases were analyzed together, patients with positive DDX21 protein expression had both significantly longer overall survival and disease-free survival times than patients with negative DDX21 protein expression (Fig. 4a,b). However, when only the subtype of microsatellite stable (MSS) cases was analyzed, the patients with positive or negative DDX21 protein expression did not display significant differences in either overall survival or disease-free survival times (Fig. 4c,d). In contrast, among patients with the (microsatellite instability) MSI subtype of early stage colorectal cancer, disease-free survival times (but not overall survival times) were significantly longer for the DDX21 positive group (Fig. 4e,f).

Among the 91 late stage patients with evaluable survival data, no significant differences in overall survival or disease-free survival times were observed between the positive and negative DDX21 protein expression groups (Fig. 5a,b). When the MSS subtype was analyzed separately, there were also no significant differences in survival times between the positive and negative DDX21 groups (Fig. 5c,d). As there were only 12 patients with the late stage MSI cancer, isolated statistical survival analyses were not meaningful for this subtype but visual inspection of survival curves did not reveal any difference.

**Figure 6:**
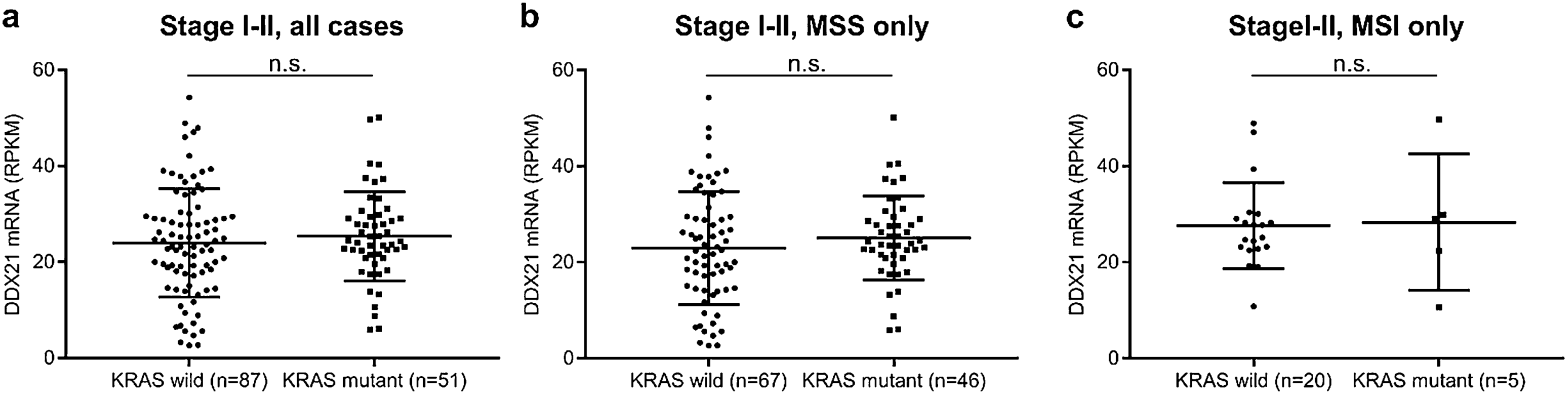
Comparison of *DDX21* gene expression levels for early stage (stages I and II) CRC stratified by *KRAS* gene status (wild type vs. mutant) using the TCGA CRC cohort (n.s., difference not significant). (**a**) Comparison for all early stage cases. (**b**) Comparison for MSS cases only. (**c**) Comparison for MSI cases only. Horizontal lines show mean (long lines) ± 1 SD (short lines).

We next asked whether there may be an association between *DDX21* expression level and *KRAS* gene status that might contribute to survival differences. Using the TCGA CRC dataset^16^, we found no differences in *DDX21* expression between early stage patients that had cancers with wild type *KRAS* vs. mutated *KRAS* (Fig. 6).

The survival analyses thus far indicated that positive DDX21 expression may be a prognostic for better disease-free survival in early stage colorectal cancer of the MSI subtype. To further explore this conclusion, we performed univariate and multivariate analyses for this group of patients (Table 3). As expected, patient age (≤ 65 years) was found to be a favorable factor for disease-free survival. In addition to patient age, positive DDX21 protein expression in cancer tissue was also an independent favorable prognostic factor for disease-free survival time in the MSI subtype of early stage colorectal cancer.

**Table 3:** Univariate and multivariate analyses of early stage MSI colorectal cancer (UICC/AJCC stages I and II combined).

Variable	Disease-free survival			
	Univariate*		Multivariate*	
	HR (95% CI)	p value	HR (95% CI)	p value
Gender (male vs. female)	0.78 (0.44–1.36)	0.3865		
Age (> 65 vs. ≤ 65)	3.30 (1.75–6.79)	0.0001	3.60 (1.90–7.42)	< 0.0001
Tumor location (right vs. left)	1.57 (0.77–3.65)	0.2236		
Histology (mucinous vs. others)	0.44 (0.15–1.01)	0.0547		
Tumor differentiation (G3 vs. G1/G2)	1.13 (0.57–2.12)	0.7057		
UICC/AJCC stage (II vs. I)	1.37 (0.78–2.50)	0.2764		
DDX21 expression (positive vs. negative)	0.44 (0.18–0.92)	**0.0274**	0.38 (0.16–0.79)	**0.0085**

p values for DDX21 < 0.05 are indiacted in bold

HR, hazard ratio; CI, confidence interval.

*Cox proportional hazards model.

## Discussion

In this study, we first used unbiased deep proteomics to identify proteome signatures that quantitatively differentiate colorectal cancer from benign colonic mucosa (Fig. 1). Specifically focusing on candidate proteins that have known antigenic properties, we selected DDX21 and investigated the protein expression of DDX21 in a large cohort of 619 patients with early stage colorectal cancer and a small cohort of 91 patients with late stage colorectal cancer (Figs. 2, 3). We found high DDX21 protein expression in about 20–30% of colorectal cancer cases, regardless of cancer stage (Tables 1, 2).

Our findings are compatible with previous reports of increased DDX21 expression in colorectal tumors at both the mRNA level and the protein levels^25,26^. The *DDX21* gene has also been found to be overexpressed in other malignancies such as breast cancer^27,28^ and lymphomas^29^. In breast cancer, *DDX21* gene expression levels have been reported to be associated with longer overall and disease-free survival^30^. In contrast, based on data in the Human Protein Atlas database (www.proteinatlas.org) and *DDX21* mRNA expression levels in a 597-case TCGA colorectal cancer cohort^17^, reduced *DDX21* mRNA transcript expression correlated with higher probability of survival, although this did not appear to be an independent prognostic marker for colorectal cancer. To reconcile this with our findings, several facts need to be considered. First, there is a major difference in that the TCGA cohort study examined the transcriptional mRNA expression of *DDX21*, whereas our study looked directly at the protein expression of DDX21. Second, quantitative levels of an mRNA transcript and its translated actual protein product do often not correlate, as has been observed for multiple tumor types in recent proteogenomic studies by the NCI’s Clinical Proteomic Tumor Analysis Consortium (CPTAC)^31–35^. Third, the TGGA cohort comprises a mixed cohort of colorectal cancer cases, whereas our outcome analyses are based on more focused cohorts, such as early stage cancer and the MSI subtype.

Cancer and the immune defense system are in a constant battle. The rising success of immunotherapy has changed the paradigm of cancer treatment from killing tumors directly to manipulating the immune system to target tumors. Most notably, immune checkpoint inhibitors have been successful in treating various malignancies, including melanoma, lung cancer, renal cell carcinoma, bladder cancer, head and neck squamous cell carcinoma, gastric cancer, ovarian cancer, Hodgkin lymphoma, and colorectal cancer^36,37^. Immune checkpoint inhibitors are effective in eliminating cancers because they can unleash the immune system to launch a broad spectrum of autoimmune-like attacks against tumors, or they may boost cancer-specific autoimmunity that is already pre-existing but weak in the cancer patients. Identification of cancer-specific autoantigens, which serve as targets of cancer-specific immunity, is instrumental for better understanding of cancer immunotherapy and therapy risk-stratification^9,10^.

DDX21, also termed nucleolar RNA helicase 2, is a known autoantigen, with autoantibodies found in patients with connective diseases and gastric antral vascular ectasia (watermelon stomach disease)^18–20^. DDX21 plays multifaceted roles in multiple steps of ribosome biogenesis and coordinates transcription and ribosomal RNA processing^38–41^. DDX21 can efficiently unwind R-loops (three-stranded nucleic acid complex consisting of an RNA:DNA heteroduplex) and prevent R-loop-mediated stalling of RNA polymerases, whereas depletion of DDX21 leads to accumulation of cellular R-loops and DNA damage^42,43^. These observations suggest that DDX21 plays important roles in cancer cell biology, although the mechanisms are yet to be defined.

Our study found that high DDX21 protein expression in cancer tissue predicts better survival for early stage colorectal cancer patients of the MSI subtype, but not for the MSS subtype or late stage cancers (Figs. 4, 5, Table 3). *DDX21* expression appears to be uncoupled from *KRAS* mutation status (Fig. 6). Among colorectal cancers, only the MSI subset has shown positive response to immune checkpoint inhibitor therapy^44–46^. However, a reliable protein response predictor of immunotherapy is still lacking. High expression of DDX21, a known nuclear autoantigen^2,19,20^, in MSI tumors may induce DDX21-specific autoimmunity against tumor cells that contributes to better clinical outcomes in cancer patients and perhaps better response to immune checkpoint inhibitor therapy. Although the latter of which will require further clinical investigation, a routine DDX21 IHC test of early stage CRCs may provide better risk prediction for MSI patients and possibly stratification of high-risk patients for adjuvant immunotherapy. Interestingly, in another recent study of ours, we found that high expression of Maspin, an autoantigen, was also associated with better clinical outcomes in the MSI subtype colorectal cancer^11^. It is possible that high expression of autoantigens in MSI cancer tissue helps induce cancer-specific autoimmunity, which consequently leads to better patient survival. It is also possible that this natural cancer-specific autoimmunity is boosted by immune checkpoint inhibitory therapies, which may lead to improved therapeutic responses.

In summary, we identified DDX21 autoantigen as a potential prognostic marker for the MSI subtype of early stage colorectal cancer. The mechanistic roles of DDX21 in cancers remain poorly understood and merit further investigation. Identification of DDX21 and other autoantigen markers in cancer tissues may pave the way for future development of more specific and more effective immunotherapy strategies against cancer.

## Supplementary Information

Supplementary Figure 1.

## Abbreviations

CRC: Colorectal cancer
DDX21: DEAD-box RNA helicase 21
DFS: Disease-free survival
IHC: Immunohistochemistry
MMR: Mismatch repair
MSS: Microsatellite stability
MSI: Microsatellite instability
OS: Overall survival

## References

[1] Guinney J (2015). The consensus molecular subtypes of colorectal cancer. Nat. Med..

[2] Dienstmann R, Salazar R, Tabernero J (2015). Personalizing colon cancer adjuvant therapy: Selecting optimal treatments for individual patients. J. Clin. Oncol..

[3] Koncina E, Haan S, Rauh S, Letellier E (2020). Prognostic and predictive molecular biomarkers for colorectal cancer: Updates and challenges. Cancers (Basel)..

[4] Mondaca S, Yaeger R (2018). Colorectal cancer genomics and designing rational trials. Ann. Transl. Med..

[5] Kaur A (2019). Immune-related adverse events in cancer patients treated with immune checkpoint inhibitors: A single-center experience. Medicine (Baltimore).

[6] Maurice C (2015). Subacute CNS demyelination after treatment with nivolumab for melanoma. Cancer Immunol. Res..

[7] Tanaka A (2020). STAT1 as a potential prognosis marker for poor outcomes of early stage colorectal cancer with microsatellite instability. PLoS ONE.

[8] Tanaka A (2020). Prolyl 4-hydroxylase alpha 1 protein expression risk-stratifies early stage colorectal cancer. Oncotarget.

[9] Yang Q, Bavi P, Wang JY, Roehrl MH (2017). Immuno-proteomic discovery of tumor tissue autoantigens identifies olfactomedin 4, CD11b, and integrin alpha-2 as markers of colorectal cancer with liver metastases. J. Proteomics.

[10] Yang Q, Roehrl MH, Wang JY (2018). Proteomic profiling of antibody-inducing immunogens in tumor tissue identifies PSMA1, LAP3, ANXA3, and maspin as colon cancer markers. Oncotarget.

[11] Tanaka A (2020). Maspin as a prognostic marker for early stage colorectal cancer with microsatellite instability. Front. Oncol..

[12] Cox J, Mann M (2008). MaxQuant enables high peptide identification rates, individualized p.p.b.-range mass accuracies and proteome-wide protein quantification. Nat. Biotechnol..

[13] Cox J (2011). Andromeda: A peptide search engine integrated into the MaxQuant environment. J. Proteome Res..

[14] Tusher VG, Tibshirani R, Chu G (2001). Significance analysis of microarrays applied to the ionizing radiation response. Proc. Natl. Acad. Sci. U.S.A..

[15] Tyanova S (2016). The Perseus computational platform for comprehensive analysis of (prote)omics data. Nat. Methods.

[16] Comprehensive molecular characterization of human colon and rectal cancer. *Nature* **487**, 330–337. 10.1038/nature11252 (2012). 10.1038/nature11252 PMC3401966 22810696

[17] Liu J (2018). An integrated TCGA pan-cancer clinical data resource to drive high-quality survival outcome analytics. Cell.

[18] Arnett FC, Reveille JD, Valdez BC (1997). Autoantibodies to a nucleolar RNA helicase protein in patients with connective tissue diseases. Arthritis Rheum..

[19] Garcia MC, Zhou J, Henning D, Arnett FC, Valdez BC (2000). Unique epitopes in RNA helicase II/Gu protein recognized by serum from a watermelon stomach patient. Mol. Immunol..

[20] Valdez BC (1996). A nucleolar RNA helicase recognized by autoimmune antibodies from a patient with watermelon stomach disease. Nucleic Acids Res..

[21] Rho JH, Zhang W, Murali M, Roehrl MH, Wang JY (2011). Human proteins with affinity for dermatan sulfate have the propensity to become autoantigens. Am. J. Pathol..

[22] Wang JY, Lee J, Yan M, Rho JH, Roehrl MH (2011). Dermatan sulfate interacts with dead cells and regulates CD5(+) B-cell fate: Implications for a key role in autoimmunity. Am. J. Pathol..

[23] Zhang W, Rho JH, Roehrl MH, Wang JY (2019). A comprehensive autoantigen-ome of autoimmune liver diseases identified from dermatan sulfate affinity enrichment of liver tissue proteins. BMC Immunol..

[24] Zhang W, Rho JH, Roehrl MW, Roehrl MH, Wang JY (2019). A repertoire of 124 potential autoantigens for autoimmune kidney diseases identified by dermatan sulfate affinity enrichment of kidney tissue proteins. PLoS ONE.

[25] Jung Y (2011). Clinical validation of colorectal cancer biomarkers identified from bioinformatics analysis of public expression data. Clin. Cancer Res..

[26] Vasaikar S (2019). Proteogenomic analysis of human colon cancer reveals new therapeutic opportunities. Cell.

[27] Zhang H (2018). A double-negative feedback loop between DEAD-box protein DDX21 and Snail regulates epithelial–mesenchymal transition and metastasis in breast cancer. Cancer Lett..

[28] Zhang Y, Baysac KC, Yee LF, Saporita AJ, Weber JD (2014). Elevated DDX21 regulates c-Jun activity and rRNA processing in human breast cancers. Breast Cancer Res..

[29] Bonzheim I (2013). Identification of C/EBPbeta target genes in ALK+ anaplastic large cell lymphoma (ALCL) by gene expression profiling and chromatin immunoprecipitation. PLoS ONE.

[30] Cimino D (2008). Identification of new genes associated with breast cancer progression by gene expression analysis of predefined sets of neoplastic tissues. Int. J. Cancer.

[31] Clark DJ (2019). Integrated proteogenomic characterization of clear cell renal cell carcinoma. Cell.

[32] Wu P (2019). Integration and analysis of CPTAC proteomics data in the context of cancer genomics in the cBioPortal. Mol. Cell Proteomics.

[33] Wang J (2017). Proteome profiling outperforms transcriptome profiling for coexpression based gene function prediction. Mol. Cell Proteomics.

[34] Mertins P (2016). Proteogenomics connects somatic mutations to signalling in breast cancer. Nature.

[35] Dou Y (2020). Proteogenomic characterization of endometrial carcinoma. Cell.

[36] Stenzel PJ (2019). Prognostic and predictive value of tumor-infiltrating leukocytes and of immune checkpoint molecules PD1 and PDL1 in clear cell renal cell carcinoma. Transl. Oncol..

[37] Kennedy LB, Salama AKS (2020). A review of cancer immunotherapy toxicity. CA Cancer J. Clin..

[38] Henning D, So RB, Jin R, Lau LF, Valdez BC (2003). Silencing of RNA helicase II/Gualpha inhibits mammalian ribosomal RNA production. J. Biol. Chem..

[39] Yang H (2003). Down-regulation of RNA helicase II/Gu results in the depletion of 18 and 28 S rRNAs in Xenopus oocyte. J. Biol. Chem..

[40] Rocak S, Linder P (2004). DEAD-box proteins: The driving forces behind RNA metabolism. Nat. Rev. Mol. Cell Biol..

[41] Calo E (2015). RNA helicase DDX21 coordinates transcription and ribosomal RNA processing. Nature.

[42] Xing YH (2017). SLERT regulates DDX21 rings associated with Pol I transcription. Cell.

[43] Song C, Hotz-Wagenblatt A, Voit R, Grummt I (2017). SIRT7 and the DEAD-box helicase DDX21 cooperate to resolve genomic R loops and safeguard genome stability. Genes Dev..

[44] Lichtenstern CR, Ngu RK, Shalapour S, Karin M (2020). Immunotherapy, inflammation and colorectal cancer. Cells.

[45] Morse MA, Hochster H, Benson A (2020). Perspectives on treatment of metastatic colorectal cancer with immune checkpoint inhibitor therapy. Oncologist.

[46] Ganesh K (2019). Immunotherapy in colorectal cancer: Rationale, challenges and potential. Nat. Rev. Gastroenterol. Hepatol..

