# Supplementary material for: DEAD-box RNA helicase protein DDX21 as a prognosis marker for early stage colorectal cancer with microsatellite instability
Source: Sci Rep. 2020 Dec 16;10:22085. doi: 10.1038/s41598-020-79049-9 (PMC7745018; doi:10.1038/s41598-020-79049-9)

Supplementary Figure 1. Tumor infiltrating lymphocyte densities (expressed as number of cells per 10 high-power fields (40× objective)) early-stage (a-c) and late-stage CRCs (d-f) and separately analyzed for combined MSS/MSI subtypes (a, d), MSS only (b, e), or MSI only (c, f). Comparisons of pairwise differences are not statistically significant (n.s.).

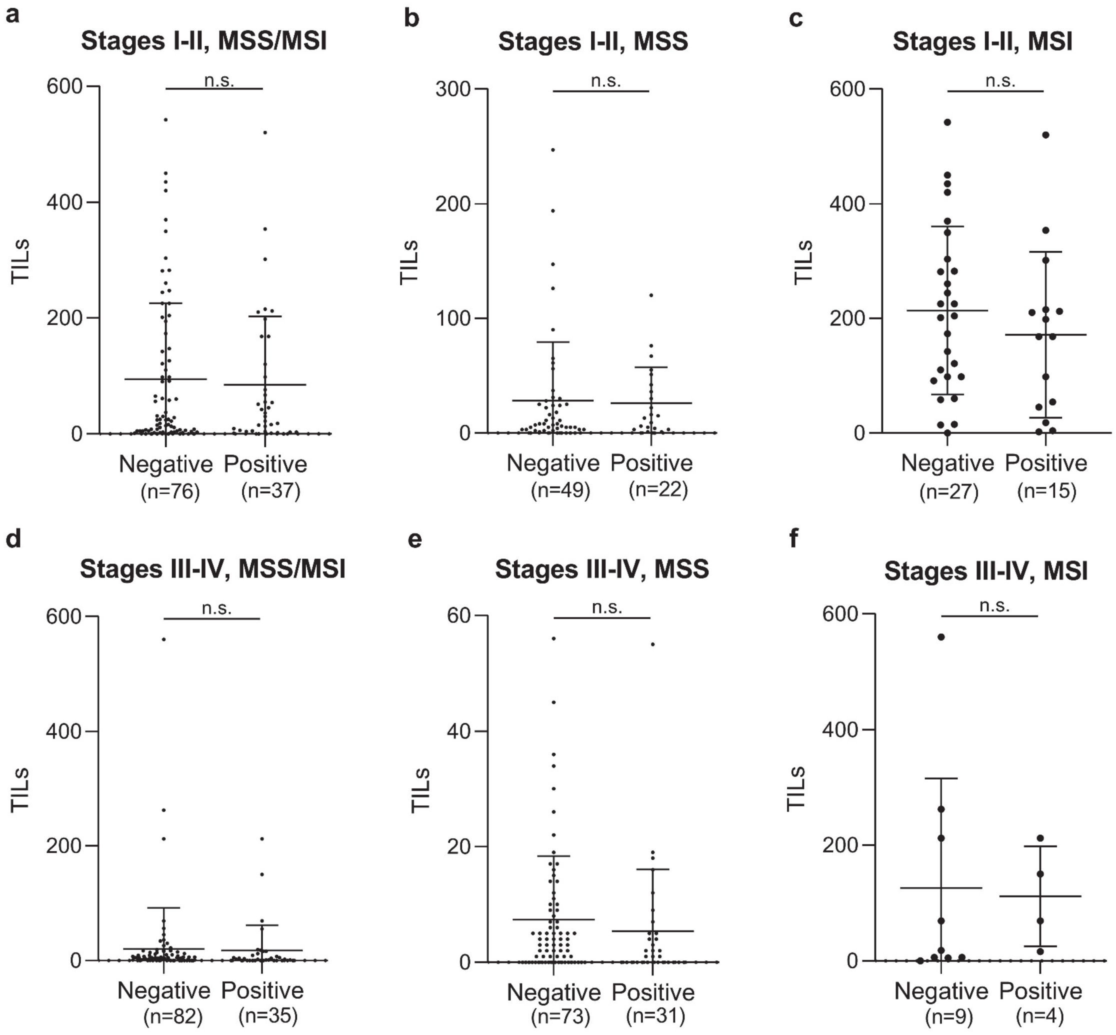

Supplement: Supplementary file 1 — Supplementary Figure 1. [file 41598_2020_79049_MOESM1_ESM.pdf]
